# Chloridobis(1,10-phenanthroline)zinc(II) tetra­chlorido(1,10-phenan­throline)bis­muthate(III) monohydrate

**DOI:** 10.1107/S1600536810052682

**Published:** 2010-12-24

**Authors:** Chunlei Song, Wenxiang Chai, Li Song, Yunyun Yang, Jian Lin

**Affiliations:** a College of Materials Science and Engineering, China Jiliang University, Hangzhou 310018, People’s Republic of China; b Department of Chemistry, Key Laboratory of Advanced Textile Materials and Manufacturing Technology of the Education Ministry, Zhejiang Sci-Tech University, Hangzhou 310018, People’s Republic of China

## Abstract

In the crystal structure of the title monohydrate salt, [ZnCl(C_12_H_8_N_2_)_2_][BiCl_4_(C_12_H_8_N_2_)]·H_2_O, the ionic components are linked into three-dimensional supra­molecular channels by five pairs of C—H⋯Cl hydrogen bonds and π–π stacking inter­actions with an inter­planar distance of 3.643 (2) Å. The solvent water mol­ecules are lodged in the channels.

## Related literature

For related bis­muth compounds, see: James *et al.* (2000 ▶); Jarraya *et al.* (1995 ▶); Bowmaker *et al.* (1998 ▶). For a related [Zn(phen)_2_Cl]^+^ coordinated cation structure, see: Yu & Zhang (2006 ▶). For supra­molecular systems containing halometallate groups as their main component, see: Mitzi & Brock (2001 ▶); Zhu *et al.* (2003 ▶); Papavassiliou *et al.* (1995 ▶); Pohl *et al.* (1994 ▶); Carmalt *et al.* (1995 ▶). For π–π inter­actions, see: Chandrasekhar *et al.* (2006 ▶). For hydrogen bonds, see: Desiraju & Steiner (1999 ▶).
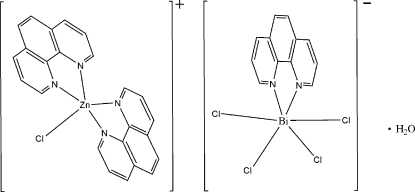


.

## Experimental

### 

#### Crystal data

[ZnCl(C_12_H_8_N_2_)_2_][BiCl_4_(C_12_H_8_N_2_)]·H_2_O
*M* *_r_* = 1010.25
Triclinic, 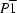

*a* = 9.748 (2) Å
*b* = 13.694 (4) Å
*c* = 14.249 (4) Å
α = 86.848 (7)°
β = 74.660 (5)°
γ = 80.692 (7)°
*V* = 1810.0 (8) Å^3^
*Z* = 2
Mo *K*α radiation
μ = 5.93 mm^−1^
*T* = 293 K
0.40 × 0.30 × 0.30 mm

#### Data collection

Rigaku R-AXIS RAPID diffractometer
Absorption correction: multi-scan (*ABSCOR*; Higashi, 1995 ▶) *T*_min_ = 0.200, *T*_max_ = 0.269
13923 measured reflections
8140 independent reflections
7571 reflections with *I* > 2σ(*I*)
*R*_int_ = 0.012

#### Refinement

*R*[*F* ^2^ > 2σ(*F* ^2^)] = 0.024
*wR*(*F* ^2^) = 0.061
*S* = 1.03
8140 reflections
451 parameters
H-atom parameters constrained
Δρ_max_ = 1.76 e Å^−3^
Δρ_min_ = −1.03 e Å^−3^

### 

Data collection: *PROCESS-AUTO* (Rigaku, 1998 ▶); cell refinement: *PROCESS-AUTO*; data reduction: *CrystalStructure* (Rigaku/MSC, 2004 ▶); program(s) used to solve structure: *SHELXS97* (Sheldrick, 2008 ▶); program(s) used to refine structure: *SHELXL97* (Sheldrick, 2008 ▶); molecular graphics: *SHELXTL* (Sheldrick, 2008 ▶); software used to prepare material for publication: *SHELXTL* and *PLATON* (Spek, 2009 ▶).

## Supplementary Material

Crystal structure: contains datablocks I, global. DOI: 10.1107/S1600536810052682/bg2383sup1.cif

Structure factors: contains datablocks I. DOI: 10.1107/S1600536810052682/bg2383Isup2.hkl

Additional supplementary materials:  crystallographic information; 3D view; checkCIF report

## Figures and Tables

**Table 1:** Hydrogen-bond geometry (Å, °)

*D*—H⋯*A*	*D*—H	H⋯*A*	*D*⋯*A*	*D*—H⋯*A*
C2—H2⋯Cl3^i^	0.93	2.82	3.588 (4)	141
C6—H6⋯Cl4^ii^	0.93	2.82	3.637 (4)	147
C10—H10⋯Cl5^iii^	0.93	2.80	3.707 (4)	164
C15—H15⋯Cl1^iv^	0.93	2.69	3.579 (4)	160
C25—H25⋯Cl2^v^	0.93	2.80	3.506 (4)	134

Symmetry codes: (i) 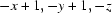; (ii) 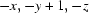; (iii) 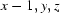; (iv) 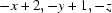; (v) 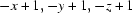.

**Fig. 1.**
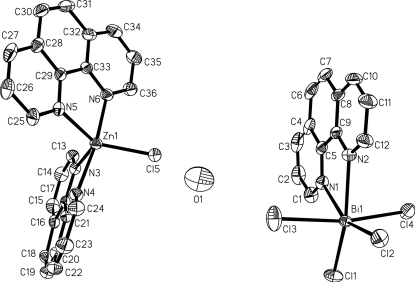
A view of the structure of I, showing the atom-numbering scheme. Displacement ellipsoids are drawn at the 30% probabilithy level and H atoms are omitted for clarity.

**Fig. 2.**
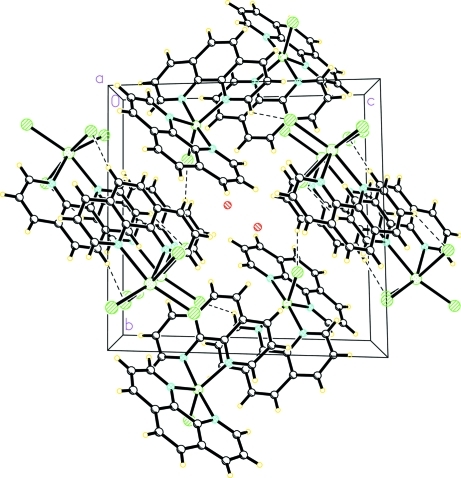
A packing diagram for I. The view shows a three-dimensional supramolecular channel along the a axis. The H atoms are shown as small spheres of arbitrary radii, and hydrogen bonds are indicated by dashed lines.

## supplementary crystallographic information

### Comment

Supramolecular compounds are attractting much interest due to their importance for the study of biological systems and their potential applications in material research, as sensors, gas storage and catalysis, or as optoelectronic and magnetic devices. Recently, many supramolecular systems containing halometallate groups as their main component have been reported (Mitzi *et al.*, 2001; Zhu *et al.*, 2003; Papavassiliou *et al.*, 1995; Pohl *et al.*, 1994; Carmalt *et al.*, 1995). Here, we present one of those supramolecular compounds [Zn(phen)_2_Cl][Bi(phen)Cl_4_].H_2_O (I), composed of an halometallate main anionic group ( [Bi(phen)Cl_4_]^- ^, phen = C_12_H_8_N_2_ = 1,10-phenanthroline), a coordinated cation containing a transition-metal, ( [Zn(phen)_2_Cl]^+^ ) and a solvent H_2_O.

In compound (I), the Bi atom is located in a distorted octahedral enviroment of four chlorine atoms and two nitrogen atoms from the phen ligand. In this BiN_2_Cl_4_ octahedron, the Bi1—N1 = 2.505 (3) Å, Bi1—N2 = 2.474 (3) Å, Bi1—Cl1 = 2.7272 (10) Å, Bi1—Cl2 = 2.6708 (10) Å, Bi1—Cl3 = 2.7841 (12) Å, Bi1—Cl4 = 2.5853 (11) Å. All bond lengths are within commonly accepted values in the literature (James *et al.*, 2000; Jarraya *et al.*, 1995). The crystal structure of the [Bi(phen)I_4_]^-^ salt has already been determined (Bowmaker *et al.*, 1998), and the Bi atom therein is coordinated in a similar distorted octahedron by two N atoms and four I atoms.

The axial and equatorial I—Bi—I bond angles therein are 165.81 (3) and 111.59 (5)° as compared to Cl3—Bi1—Cl4 = 169.50 (4) and Cl1—Bi1—Cl2 = 117.18 (4)° , respectively. The large deviations of these bond angles from those in the perfect octahedron are probably derived from the inert electron pair effect of the Bi atom. A [Zn(phen)_2_Cl]^+^ cation balances charge in the salt. This coordinated cation has been reported elsewhere (Yu *et al.*, 2006), with the Zn atom also located in a distorted trigonal-bipyramidal coordination.

In the crystal structure of I, hydrogen bonds and offset face-to face aromatic π-π stacking interactions lead to the formation of a three-dimensional supramolecular channel, and the solvent water molecules are located within. Firstly, the [Bi(phen)Cl_4_]^-^ anion and [Zn(phen)_2_Cl]^+^ cations connect to each other by hydrogen bonding interactions (details listed in Table 1), and the result is the building up a supramolecular sheet. The hydrogen bonding data are in the normal range (Desiraju *et al.*, 1999). Adjacent sheets are joined together by way of π-π stacking interactions between two phen ligands to form a three-dimensional framework (Chandrasekhar *et al.*, 2006). The phen skeletons are arranged in a parallel fashion; ring 1 (N5/C25—C29) [symmetry code: (*x*, *y*, *z*)] of one cation stacks with ring 2 (C28—C33) [symmetry code: (1 - *x*, -*y*, 1 - *z*)] of a neighbouring cation with an interplanar distance of 3.643 (2) Å. As a result, through these π-π stacking interactions, the supramolecular sheets stack one by one to present a firm three-dimensional supramolecular channel, where the water molecules are located. Even if the water hydrogens could not be determined in the difference Fourier, the geometry around O1 strongly suggests H-bonding interactions between O1 and the neighbouring Cl atoms (O1···Cl3 : 3.3723 (7) Å ; O1···Cl5: 3.3776 (6) Å ).

### Experimental

The title compound (I) was synthesized by hydrothermal reaction of ZnCl_2_ (136 mg, 1 mmol), Bi(NO_3_)_3_.5H_2_O (250 mg, 0.52 mmol), oxalic acid (380 mg, 3 mmol) and 1,10-phenanthroline monohydrate (400 mg, 2 mmol) in 5 mL water. The mixture was heated to 393 K at the rate of 20 K/h, and kept at this temperature for 2 days and then cooled to room temperature at the rate of 2 K/h. The yellow crystals of (I) were obtained in a yield of 18% (73 mg). Anal. Calc. for C_36_H_26_BiCl_5_N_6_OZn (%): C, 42.80; H, 2.59; N, 8.32; O, 1.58. Found: C, 42.96; H, 2.77; N, 8.23;O, 1.74. Crystals of (I) suitable for single-crystal X-ray diffraction were selected directly from the sample as prepared.

### Refinement

All hydrogen atoms attached to C were added at calculated positions and refined using a riding model, (C-H: . Due to the presence of Bi in the structure, those pertaining to the hydration water O1 could not be found in the difference Fourier map and were not included in the model.

### Crystal data

**Table:**

[ZnCl(C_12_H_8_N_2_)_2_][BiCl_4_(C_12_H_8_N_2_)]·H_2_O	*Z* = 2
*M**_r_* = 1010.25	*F*(000) = 980
Triclinic, *P*1	*D*_x_ = 1.854 Mg m^−^^3^
Hall symbol: -P 1	Mo *K*α radiation, λ = 0.71075 Å
*a* = 9.748 (2) Å	Cell parameters from 5205 reflections
*b* = 13.694 (4) Å	θ = 2.1–27.5°
*c* = 14.249 (4) Å	µ = 5.93 mm^−^^1^
α = 86.848 (7)°	*T* = 293 K
β = 74.660 (5)°	Block, yellow
γ = 80.692 (7)°	0.40 × 0.30 × 0.30 mm
*V* = 1810.0 (8) Å^3^	

### Data collection

**Table:**

Rigaku R-AXIS RAPID diffractometer	8140 independent reflections
Radiation source: fine-focus sealed tube	7571 reflections with *I* > 2σ(*I*)
graphite	*R*_int_ = 0.012
Detector resolution: 14.6306 pixels mm^-1^	θ_max_ = 27.5°, θ_min_ = 2.1°
CCD_Profile_fitting scans	*h* = −12→12
Absorption correction: multi-scan (*ABSCOR*; Higashi, 1995)	*k* = −17→17
*T*_min_ = 0.200, *T*_max_ = 0.269	*l* = −18→15
13923 measured reflections	

### Refinement

**Table:**

Refinement on *F*^2^	Primary atom site location: structure-invariant direct methods
Least-squares matrix: full	Secondary atom site location: difference Fourier map
*R*[*F*^2^ > 2σ(*F*^2^)] = 0.024	Hydrogen site location: inferred from neighbouring sites
*wR*(*F*^2^) = 0.061	H-atom parameters constrained
*S* = 1.03	*w* = 1/[σ^2^(*F*_o_^2^) + (0.035*P*)^2^ + 0.8909*P*] where *P* = (*F*_o_^2^ + 2*F*_c_^2^)/3
8140 reflections	(Δ/σ)_max_ = 0.003
451 parameters	Δρ_max_ = 1.76 e Å^−^^3^
0 restraints	Δρ_min_ = −1.03 e Å^−^^3^

### Special details

**Table:**

Geometry. All e.s.d.'s (except the e.s.d. in the dihedral angle between two l.s. planes) are estimated using the full covariance matrix. The cell e.s.d.'s are taken into account individually in the estimation of e.s.d.'s in distances, angles and torsion angles; correlations between e.s.d.'s in cell parameters are only used when they are defined by crystal symmetry. An approximate (isotropic) treatment of cell e.s.d.'s is used for estimating e.s.d.'s involving l.s. planes.
Refinement. Refinement of *F*^2^ against ALL reflections. The weighted *R*-factor *wR* and goodness of fit *S* are based on *F*^2^, conventional *R*-factors *R* are based on *F*, with *F* set to zero for negative *F*^2^. The threshold expression of *F*^2^ > σ(*F*^2^) is used only for calculating *R*-factors(gt) *etc*. and is not relevant to the choice of reflections for refinement. *R*-factors based on *F*^2^ are statistically about twice as large as those based on *F*, and *R*- factors based on ALL data will be even larger.

### Fractional atomic coordinates and isotropic or equivalent isotropic displacement parameters (Å^2^)

**Table:**

	*x*	*y*	*z*	*U*_iso_*/*U*_eq_	
Bi1	0.187173 (6)	0.746413 (5)	0.167412 (4)	0.03413 (2)	
Zn1	0.78062 (2)	0.150812 (15)	0.327965 (15)	0.03602 (5)	
Cl1	0.40709 (6)	0.82384 (5)	0.04129 (4)	0.06274 (16)	
Cl3	0.35492 (8)	0.63095 (6)	0.27504 (5)	0.0836 (2)	
Cl4	0.01178 (7)	0.82443 (4)	0.06630 (5)	0.07034 (15)	
Cl5	0.62441 (5)	0.27909 (4)	0.29060 (5)	0.05442 (14)	
Cl2	0.03588 (6)	0.85536 (4)	0.32106 (4)	0.06049 (15)	
O1	0.4158 (4)	0.4571 (2)	0.4424 (2)	0.1368 (12)	
N1	0.24551 (18)	0.60304 (12)	0.05446 (12)	0.0432 (4)	
N2	0.01895 (17)	0.62449 (12)	0.21779 (12)	0.0430 (4)	
N3	0.98388 (15)	0.12811 (11)	0.23401 (11)	0.0347 (4)	
N4	0.89400 (17)	0.24614 (12)	0.39095 (12)	0.0404 (4)	
N5	0.75232 (16)	0.04934 (12)	0.44323 (11)	0.0385 (4)	
N6	0.68957 (17)	0.04085 (12)	0.27109 (11)	0.0412 (4)	
C1	0.3532 (3)	0.59510 (18)	−0.02525 (16)	0.0569 (6)	
H1	0.4077	0.6462	−0.0419	0.068*	
C2	0.3879 (3)	0.5125 (2)	−0.08527 (18)	0.0699 (8)	
H2	0.4652	0.5084	−0.1403	0.084*	
C3	0.3071 (3)	0.43848 (18)	−0.06212 (17)	0.0719 (7)	
H3	0.3304	0.3826	−0.1008	0.086*	
C4	0.1870 (3)	0.44578 (15)	0.02089 (16)	0.0568 (6)	
C5	0.1617 (2)	0.53089 (14)	0.07824 (14)	0.0428 (5)	
C6	0.0902 (3)	0.37504 (16)	0.04618 (19)	0.0721 (6)	
H6	0.1071	0.3189	0.0084	0.087*	
C7	−0.0242 (3)	0.38787 (16)	0.12303 (19)	0.0695 (6)	
H7	−0.0870	0.3415	0.1366	0.083*	
C8	−0.0520 (2)	0.47192 (15)	0.18510 (17)	0.0549 (5)	
C9	0.0414 (2)	0.54307 (14)	0.16269 (14)	0.0420 (5)	
C10	−0.1687 (3)	0.48762 (18)	0.2676 (2)	0.0683 (7)	
H10	−0.2324	0.4419	0.2848	0.082*	
C11	−0.1894 (3)	0.5695 (2)	0.3228 (2)	0.0694 (8)	
H11	−0.2667	0.5801	0.3776	0.083*	
C12	−0.0926 (2)	0.63737 (18)	0.29562 (18)	0.0554 (6)	
H12	−0.1068	0.6933	0.3332	0.067*	
C13	1.0301 (2)	0.06529 (15)	0.16107 (14)	0.0437 (5)	
H13	0.9688	0.0232	0.1521	0.052*	
C14	1.1661 (2)	0.05940 (17)	0.09739 (15)	0.0526 (6)	
H14	1.1949	0.0137	0.0473	0.063*	
C15	1.2568 (2)	0.12066 (18)	0.10860 (16)	0.0530 (6)	
H15	1.3479	0.1176	0.0659	0.064*	
C16	1.2123 (2)	0.18894 (15)	0.18524 (14)	0.0416 (5)	
C17	1.07434 (18)	0.18846 (13)	0.24784 (12)	0.0331 (4)	
C18	1.2984 (2)	0.25820 (17)	0.20192 (17)	0.0519 (6)	
H18	1.3886	0.2605	0.1594	0.062*	
C19	1.2519 (2)	0.31960 (16)	0.27741 (18)	0.0536 (6)	
H19	1.3092	0.3650	0.2854	0.064*	
C20	1.1157 (2)	0.31706 (14)	0.34626 (16)	0.0456 (5)	
C21	1.02573 (18)	0.25202 (13)	0.33039 (13)	0.0358 (4)	
C22	1.0659 (3)	0.37315 (16)	0.43134 (18)	0.0578 (6)	
H22	1.1224	0.4159	0.4458	0.069*	
C23	0.9347 (3)	0.36507 (16)	0.49287 (17)	0.0591 (6)	
H23	0.9023	0.4011	0.5501	0.071*	
C24	0.8495 (2)	0.30251 (16)	0.46956 (16)	0.0503 (6)	
H24	0.7583	0.3001	0.5104	0.060*	
C25	0.7844 (2)	0.05377 (17)	0.52733 (15)	0.0505 (6)	
H25	0.8275	0.1065	0.5380	0.061*	
C26	0.7559 (3)	−0.01784 (19)	0.60065 (17)	0.0638 (7)	
H26	0.7792	−0.0120	0.6591	0.077*	
C27	0.6945 (3)	−0.09541 (18)	0.58647 (17)	0.0606 (7)	
H27	0.6725	−0.1421	0.6359	0.073*	
C28	0.6640 (2)	−0.10539 (15)	0.49649 (16)	0.0472 (6)	
C29	0.69474 (18)	−0.03026 (13)	0.42664 (14)	0.0369 (4)	
C30	0.6035 (2)	−0.18692 (17)	0.47345 (19)	0.0613 (7)	
H30	0.5856	−0.2380	0.5187	0.074*	
C31	0.5726 (2)	−0.19043 (17)	0.3879 (2)	0.0610 (7)	
H31	0.5316	−0.2435	0.3754	0.073*	
C32	0.6008 (2)	−0.11468 (15)	0.31452 (17)	0.0482 (6)	
C33	0.66186 (18)	−0.03468 (14)	0.33480 (14)	0.0378 (5)	
C34	0.5661 (2)	−0.11203 (18)	0.22484 (18)	0.0595 (6)	
H34	0.5259	−0.1635	0.2081	0.071*	
C35	0.5913 (2)	−0.03448 (19)	0.16243 (17)	0.0596 (6)	
H35	0.5667	−0.0320	0.1035	0.071*	
C36	0.6542 (2)	0.04149 (17)	0.18741 (16)	0.0518 (6)	
H36	0.6719	0.0941	0.1441	0.062*	

### Atomic displacement parameters (Å^2^)

**Table:**

	*U*^11^	*U*^22^	*U*^33^	*U*^12^	*U*^13^	*U*^23^
Bi1	0.03586 (3)	0.03625 (3)	0.03228 (3)	−0.00975 (2)	−0.00893 (2)	−0.00470 (2)
Zn1	0.03522 (9)	0.03824 (10)	0.03538 (10)	−0.00949 (8)	−0.00859 (8)	0.00157 (8)
Cl1	0.0602 (3)	0.0771 (3)	0.0524 (3)	−0.0367 (2)	−0.0012 (2)	−0.0027 (2)
Cl3	0.0772 (4)	0.1053 (5)	0.0550 (3)	0.0289 (4)	−0.0178 (3)	−0.0103 (3)
Cl4	0.0946 (3)	0.0496 (3)	0.0893 (3)	−0.0090 (3)	−0.0637 (3)	−0.0025 (2)
Cl5	0.0441 (2)	0.0436 (2)	0.0793 (3)	−0.00778 (19)	−0.0237 (2)	0.0085 (2)
Cl2	0.0624 (3)	0.0617 (3)	0.0540 (3)	0.0005 (2)	−0.0108 (2)	−0.0227 (2)
O1	0.147 (2)	0.143 (2)	0.117 (2)	−0.033 (2)	−0.0196 (19)	−0.0161 (19)
N1	0.0500 (8)	0.0410 (8)	0.0382 (8)	−0.0010 (7)	−0.0127 (7)	−0.0069 (6)
N2	0.0451 (8)	0.0410 (8)	0.0453 (8)	−0.0138 (6)	−0.0118 (7)	0.0009 (7)
N3	0.0368 (7)	0.0334 (7)	0.0332 (7)	−0.0038 (6)	−0.0088 (6)	0.0005 (6)
N4	0.0410 (7)	0.0396 (7)	0.0414 (8)	−0.0051 (6)	−0.0114 (6)	−0.0062 (6)
N5	0.0396 (7)	0.0403 (8)	0.0351 (7)	−0.0013 (6)	−0.0119 (6)	0.0014 (6)
N6	0.0454 (7)	0.0444 (8)	0.0390 (8)	−0.0134 (6)	−0.0164 (6)	0.0018 (6)
C1	0.0560 (12)	0.0624 (13)	0.0489 (11)	0.0006 (10)	−0.0109 (10)	−0.0132 (10)
C2	0.0759 (15)	0.0763 (15)	0.0503 (12)	0.0219 (13)	−0.0187 (11)	−0.0278 (11)
C3	0.1028 (16)	0.0555 (12)	0.0625 (12)	0.0276 (12)	−0.0483 (11)	−0.0279 (10)
C4	0.0866 (12)	0.0337 (9)	0.0613 (10)	0.0111 (9)	−0.0489 (9)	−0.0104 (8)
C5	0.0569 (9)	0.0329 (8)	0.0471 (9)	−0.0016 (7)	−0.0315 (7)	−0.0014 (7)
C6	0.1195 (14)	0.0295 (9)	0.0942 (13)	0.0001 (10)	−0.0803 (11)	−0.0059 (9)
C7	0.1020 (13)	0.0343 (9)	0.1019 (14)	−0.0225 (9)	−0.0745 (11)	0.0173 (10)
C8	0.0716 (10)	0.0383 (9)	0.0753 (11)	−0.0222 (8)	−0.0511 (9)	0.0218 (8)
C9	0.0506 (9)	0.0354 (8)	0.0496 (9)	−0.0100 (7)	−0.0295 (7)	0.0094 (7)
C10	0.0674 (11)	0.0634 (12)	0.0915 (15)	−0.0389 (10)	−0.0414 (11)	0.0387 (11)
C11	0.0554 (12)	0.0791 (15)	0.0740 (16)	−0.0308 (11)	−0.0097 (12)	0.0206 (13)
C12	0.0490 (10)	0.0583 (12)	0.0583 (13)	−0.0170 (9)	−0.0080 (10)	0.0022 (10)
C13	0.0487 (9)	0.0429 (9)	0.0400 (9)	−0.0067 (8)	−0.0118 (8)	−0.0043 (8)
C14	0.0557 (11)	0.0562 (12)	0.0382 (10)	−0.0004 (10)	−0.0025 (9)	−0.0071 (9)
C15	0.0394 (10)	0.0659 (13)	0.0440 (11)	−0.0014 (10)	0.0011 (9)	0.0035 (10)
C16	0.0341 (8)	0.0483 (10)	0.0405 (9)	−0.0041 (8)	−0.0101 (7)	0.0107 (8)
C17	0.0340 (7)	0.0338 (8)	0.0329 (8)	−0.0036 (6)	−0.0134 (6)	0.0057 (6)
C18	0.0347 (8)	0.0642 (12)	0.0584 (12)	−0.0164 (8)	−0.0129 (8)	0.0159 (10)
C19	0.0462 (9)	0.0524 (11)	0.0710 (13)	−0.0209 (8)	−0.0251 (9)	0.0109 (10)
C20	0.0505 (9)	0.0366 (9)	0.0594 (11)	−0.0109 (8)	−0.0297 (8)	0.0038 (8)
C21	0.0367 (8)	0.0327 (8)	0.0414 (9)	−0.0045 (7)	−0.0171 (7)	0.0013 (7)
C22	0.0693 (11)	0.0424 (10)	0.0745 (13)	−0.0137 (9)	−0.0362 (10)	−0.0084 (9)
C23	0.0770 (14)	0.0485 (11)	0.0558 (12)	−0.0079 (10)	−0.0216 (10)	−0.0190 (9)
C24	0.0558 (11)	0.0478 (10)	0.0458 (10)	−0.0064 (9)	−0.0092 (9)	−0.0114 (8)
C25	0.0591 (11)	0.0534 (11)	0.0411 (10)	−0.0002 (9)	−0.0219 (8)	0.0015 (9)
C26	0.0781 (14)	0.0688 (15)	0.0427 (11)	0.0056 (12)	−0.0243 (10)	0.0070 (10)
C27	0.0637 (13)	0.0607 (13)	0.0459 (11)	0.0065 (11)	−0.0078 (10)	0.0189 (10)
C28	0.0383 (9)	0.0436 (10)	0.0500 (11)	0.0023 (8)	−0.0014 (8)	0.0089 (9)
C29	0.0285 (7)	0.0381 (9)	0.0391 (9)	−0.0002 (7)	−0.0036 (7)	0.0029 (7)
C30	0.0521 (11)	0.0488 (11)	0.0740 (15)	−0.0122 (10)	−0.0022 (11)	0.0196 (11)
C31	0.0511 (11)	0.0444 (10)	0.0840 (17)	−0.0184 (9)	−0.0058 (11)	0.0039 (11)
C32	0.0394 (9)	0.0416 (9)	0.0618 (12)	−0.0105 (8)	−0.0064 (9)	−0.0055 (9)
C33	0.0293 (7)	0.0401 (9)	0.0419 (9)	−0.0055 (7)	−0.0053 (7)	−0.0004 (7)
C34	0.0540 (10)	0.0629 (12)	0.0679 (13)	−0.0198 (10)	−0.0170 (10)	−0.0164 (10)
C35	0.0634 (11)	0.0718 (13)	0.0526 (11)	−0.0185 (11)	−0.0236 (9)	−0.0124 (10)
C36	0.0611 (11)	0.0574 (11)	0.0429 (10)	−0.0173 (9)	−0.0194 (9)	0.0016 (9)

### Geometric parameters (Å, °)

**Table:**

Bi1—N2	2.4745 (17)	C12—H12	0.9300
Bi1—N1	2.5041 (17)	C13—C14	1.387 (3)
Bi1—Cl4	2.5853 (8)	C13—H13	0.9300
Bi1—Cl2	2.6708 (8)	C14—C15	1.356 (4)
Bi1—Cl1	2.7271 (7)	C14—H14	0.9300
Bi1—Cl3	2.7838 (9)	C15—C16	1.409 (3)
Zn1—N3	2.0635 (14)	C15—H15	0.9300
Zn1—N5	2.0828 (16)	C16—C17	1.404 (2)
Zn1—N6	2.1586 (18)	C16—C18	1.431 (3)
Zn1—N4	2.2026 (18)	C17—C21	1.432 (2)
Zn1—Cl5	2.2690 (7)	C18—C19	1.337 (3)
N1—C1	1.323 (3)	C18—H18	0.9300
N1—C5	1.355 (3)	C19—C20	1.431 (3)
N2—C12	1.329 (3)	C19—H19	0.9300
N2—C9	1.354 (3)	C20—C22	1.402 (3)
N3—C13	1.322 (2)	C20—C21	1.412 (3)
N3—C17	1.358 (2)	C22—C23	1.363 (3)
N4—C24	1.330 (3)	C22—H22	0.9300
N4—C21	1.356 (2)	C23—C24	1.393 (3)
N5—C25	1.323 (3)	C23—H23	0.9300
N5—C29	1.362 (3)	C24—H24	0.9300
N6—C36	1.325 (3)	C25—C26	1.397 (3)
N6—C33	1.351 (2)	C25—H25	0.9300
C1—C2	1.398 (3)	C26—C27	1.349 (4)
C1—H1	0.9300	C26—H26	0.9300
C2—C3	1.357 (4)	C27—C28	1.409 (4)
C2—H2	0.9300	C27—H27	0.9300
C3—C4	1.422 (3)	C28—C29	1.403 (3)
C3—H3	0.9300	C28—C30	1.434 (3)
C4—C5	1.411 (3)	C29—C33	1.434 (3)
C4—C6	1.427 (3)	C30—C31	1.336 (4)
C5—C9	1.436 (3)	C30—H30	0.9300
C6—C7	1.336 (4)	C31—C32	1.437 (3)
C6—H6	0.9300	C31—H31	0.9300
C7—C8	1.437 (3)	C32—C34	1.403 (4)
C7—H7	0.9300	C32—C33	1.405 (3)
C8—C10	1.401 (3)	C34—C35	1.358 (4)
C8—C9	1.407 (3)	C34—H34	0.9300
C10—C11	1.363 (4)	C35—C36	1.396 (3)
C10—H10	0.9300	C35—H35	0.9300
C11—C12	1.397 (3)	C36—H36	0.9300
C11—H11	0.9300		
			
N2—Bi1—N1	66.87 (6)	N2—C12—C11	122.1 (2)
N2—Bi1—Cl4	84.27 (5)	N2—C12—H12	119.0
N1—Bi1—Cl4	85.90 (5)	C11—C12—H12	119.0
N2—Bi1—Cl2	88.88 (4)	N3—C13—C14	122.8 (2)
N1—Bi1—Cl2	155.73 (4)	N3—C13—H13	118.6
Cl4—Bi1—Cl2	91.03 (3)	C14—C13—H13	118.6
N2—Bi1—Cl1	153.56 (4)	C15—C14—C13	119.6 (2)
N1—Bi1—Cl1	86.95 (4)	C15—C14—H14	120.2
Cl4—Bi1—Cl1	90.49 (3)	C13—C14—H14	120.2
Cl2—Bi1—Cl1	117.18 (2)	C14—C15—C16	119.66 (18)
N2—Bi1—Cl3	86.01 (5)	C14—C15—H15	120.2
N1—Bi1—Cl3	86.63 (5)	C16—C15—H15	120.2
Cl4—Bi1—Cl3	169.50 (2)	C17—C16—C15	117.14 (19)
Cl2—Bi1—Cl3	92.75 (3)	C17—C16—C18	118.88 (18)
Cl1—Bi1—Cl3	96.48 (3)	C15—C16—C18	123.98 (18)
N3—Zn1—N5	113.48 (6)	N3—C17—C16	122.25 (16)
N3—Zn1—N6	98.12 (6)	N3—C17—C21	117.79 (15)
N5—Zn1—N6	78.81 (7)	C16—C17—C21	119.95 (17)
N3—Zn1—N4	78.42 (6)	C19—C18—C16	121.28 (18)
N5—Zn1—N4	96.10 (7)	C19—C18—H18	119.4
N6—Zn1—N4	172.24 (6)	C16—C18—H18	119.4
N3—Zn1—Cl5	116.25 (5)	C18—C19—C20	121.5 (2)
N5—Zn1—Cl5	130.27 (4)	C18—C19—H19	119.3
N6—Zn1—Cl5	93.82 (5)	C20—C19—H19	119.3
N4—Zn1—Cl5	93.95 (5)	C22—C20—C21	116.82 (18)
C1—N1—C5	119.74 (18)	C22—C20—C19	124.3 (2)
C1—N1—Bi1	123.11 (15)	C21—C20—C19	118.81 (19)
C5—N1—Bi1	117.14 (12)	N4—C21—C20	122.70 (17)
C12—N2—C9	119.59 (18)	N4—C21—C17	117.84 (17)
C12—N2—Bi1	122.15 (15)	C20—C21—C17	119.46 (16)
C9—N2—Bi1	118.25 (12)	C23—C22—C20	120.0 (2)
C13—N3—C17	118.48 (15)	C23—C22—H22	120.0
C13—N3—Zn1	126.69 (14)	C20—C22—H22	120.0
C17—N3—Zn1	114.75 (11)	C22—C23—C24	119.6 (2)
C24—N4—C21	118.43 (18)	C22—C23—H23	120.2
C24—N4—Zn1	131.04 (14)	C24—C23—H23	120.2
C21—N4—Zn1	110.17 (12)	N4—C24—C23	122.4 (2)
C25—N5—C29	118.35 (17)	N4—C24—H24	118.8
C25—N5—Zn1	127.75 (15)	C23—C24—H24	118.8
C29—N5—Zn1	113.89 (13)	N5—C25—C26	122.4 (2)
C36—N6—C33	119.11 (18)	N5—C25—H25	118.8
C36—N6—Zn1	128.84 (14)	C26—C25—H25	118.8
C33—N6—Zn1	111.98 (13)	C27—C26—C25	119.9 (2)
N1—C1—C2	122.3 (2)	C27—C26—H26	120.1
N1—C1—H1	118.9	C25—C26—H26	120.1
C2—C1—H1	118.9	C26—C27—C28	119.7 (2)
C3—C2—C1	119.1 (2)	C26—C27—H27	120.2
C3—C2—H2	120.5	C28—C27—H27	120.2
C1—C2—H2	120.5	C29—C28—C27	117.1 (2)
C2—C3—C4	120.4 (2)	C29—C28—C30	119.1 (2)
C2—C3—H3	119.8	C27—C28—C30	123.7 (2)
C4—C3—H3	119.8	N5—C29—C28	122.45 (19)
C5—C4—C3	116.5 (2)	N5—C29—C33	117.80 (16)
C5—C4—C6	119.4 (2)	C28—C29—C33	119.74 (19)
C3—C4—C6	124.0 (2)	C31—C30—C28	120.8 (2)
N1—C5—C4	121.85 (18)	C31—C30—H30	119.6
N1—C5—C9	118.84 (17)	C28—C30—H30	119.6
C4—C5—C9	119.29 (19)	C30—C31—C32	122.0 (2)
C7—C6—C4	121.3 (2)	C30—C31—H31	119.0
C7—C6—H6	119.4	C32—C31—H31	119.0
C4—C6—H6	119.4	C34—C32—C33	116.9 (2)
C6—C7—C8	121.3 (2)	C34—C32—C31	124.8 (2)
C6—C7—H7	119.4	C33—C32—C31	118.3 (2)
C8—C7—H7	119.4	N6—C33—C32	122.45 (19)
C10—C8—C9	117.5 (2)	N6—C33—C29	117.52 (17)
C10—C8—C7	123.4 (2)	C32—C33—C29	120.03 (18)
C9—C8—C7	119.1 (2)	C35—C34—C32	120.1 (2)
N2—C9—C8	121.52 (18)	C35—C34—H34	119.9
N2—C9—C5	118.83 (17)	C32—C34—H34	119.9
C8—C9—C5	119.63 (18)	C34—C35—C36	119.5 (2)
C11—C10—C8	120.3 (2)	C34—C35—H35	120.2
C11—C10—H10	119.8	C36—C35—H35	120.2
C8—C10—H10	119.8	N6—C36—C35	121.9 (2)
C10—C11—C12	119.0 (2)	N6—C36—H36	119.0
C10—C11—H11	120.5	C35—C36—H36	119.0
C12—C11—H11	120.5		
			
N2—Bi1—N1—C1	178.34 (19)	C7—C8—C10—C11	−179.4 (2)
Cl4—Bi1—N1—C1	92.83 (17)	C8—C10—C11—C12	0.1 (4)
Cl2—Bi1—N1—C1	176.22 (14)	C9—N2—C12—C11	−0.4 (4)
Cl1—Bi1—N1—C1	2.12 (17)	Bi1—N2—C12—C11	178.55 (19)
Cl3—Bi1—N1—C1	−94.56 (17)	C10—C11—C12—N2	−0.1 (4)
N2—Bi1—N1—C5	−2.14 (14)	C17—N3—C13—C14	−0.9 (3)
Cl4—Bi1—N1—C5	−87.65 (14)	Zn1—N3—C13—C14	175.64 (16)
Cl2—Bi1—N1—C5	−4.3 (2)	N3—C13—C14—C15	−0.6 (3)
Cl1—Bi1—N1—C5	−178.36 (14)	C13—C14—C15—C16	0.5 (3)
Cl3—Bi1—N1—C5	84.96 (14)	C14—C15—C16—C17	1.0 (3)
N1—Bi1—N2—C12	−178.07 (19)	C14—C15—C16—C18	−178.5 (2)
Cl4—Bi1—N2—C12	−90.09 (17)	C13—N3—C17—C16	2.6 (3)
Cl2—Bi1—N2—C12	1.06 (17)	Zn1—N3—C17—C16	−174.40 (14)
Cl1—Bi1—N2—C12	−169.56 (14)	C13—N3—C17—C21	−176.49 (17)
Cl3—Bi1—N2—C12	93.90 (17)	Zn1—N3—C17—C21	6.5 (2)
N1—Bi1—N2—C9	0.89 (14)	C15—C16—C17—N3	−2.6 (3)
Cl4—Bi1—N2—C9	88.87 (14)	C18—C16—C17—N3	176.91 (18)
Cl2—Bi1—N2—C9	−179.98 (14)	C15—C16—C17—C21	176.45 (18)
Cl1—Bi1—N2—C9	9.4 (2)	C18—C16—C17—C21	−4.1 (3)
Cl3—Bi1—N2—C9	−87.14 (14)	C17—C16—C18—C19	2.2 (3)
N5—Zn1—N3—C13	83.48 (17)	C15—C16—C18—C19	−178.3 (2)
N6—Zn1—N3—C13	2.21 (17)	C16—C18—C19—C20	1.7 (3)
N4—Zn1—N3—C13	175.16 (17)	C18—C19—C20—C22	174.2 (2)
Cl5—Zn1—N3—C13	−96.15 (16)	C18—C19—C20—C21	−3.7 (3)
N5—Zn1—N3—C17	−99.84 (13)	C24—N4—C21—C20	−1.5 (3)
N6—Zn1—N3—C17	178.89 (12)	Zn1—N4—C21—C20	172.35 (15)
N4—Zn1—N3—C17	−8.17 (12)	C24—N4—C21—C17	177.89 (18)
Cl5—Zn1—N3—C17	80.53 (13)	Zn1—N4—C21—C17	−8.2 (2)
N3—Zn1—N4—C24	−178.41 (19)	C22—C20—C21—N4	3.1 (3)
N5—Zn1—N4—C24	−65.64 (19)	C19—C20—C21—N4	−178.80 (19)
Cl5—Zn1—N4—C24	65.59 (18)	C22—C20—C21—C17	−176.29 (18)
N3—Zn1—N4—C21	8.74 (12)	C19—C20—C21—C17	1.8 (3)
N5—Zn1—N4—C21	121.51 (12)	N3—C17—C21—N4	1.7 (2)
Cl5—Zn1—N4—C21	−107.27 (12)	C16—C17—C21—N4	−177.41 (17)
N3—Zn1—N5—C25	85.42 (17)	N3—C17—C21—C20	−178.89 (17)
N6—Zn1—N5—C25	179.49 (17)	C16—C17—C21—C20	2.0 (3)
N4—Zn1—N5—C25	5.42 (16)	C21—C20—C22—C23	−1.6 (3)
Cl5—Zn1—N5—C25	−95.02 (16)	C19—C20—C22—C23	−179.5 (2)
N3—Zn1—N5—C29	−94.02 (12)	C20—C22—C23—C24	−1.4 (4)
N6—Zn1—N5—C29	0.06 (11)	C21—N4—C24—C23	−1.7 (3)
N4—Zn1—N5—C29	−174.01 (11)	Zn1—N4—C24—C23	−174.04 (17)
Cl5—Zn1—N5—C29	85.55 (12)	C22—C23—C24—N4	3.2 (4)
N3—Zn1—N6—C36	−70.90 (17)	C29—N5—C25—C26	−2.7 (3)
N5—Zn1—N6—C36	176.64 (17)	Zn1—N5—C25—C26	177.91 (16)
Cl5—Zn1—N6—C36	46.31 (17)	N5—C25—C26—C27	0.5 (3)
N3—Zn1—N6—C33	112.22 (12)	C25—C26—C27—C28	2.1 (3)
N5—Zn1—N6—C33	−0.24 (11)	C26—C27—C28—C29	−2.5 (3)
Cl5—Zn1—N6—C33	−130.57 (11)	C26—C27—C28—C30	177.8 (2)
C5—N1—C1—C2	−2.3 (3)	C25—N5—C29—C28	2.2 (3)
Bi1—N1—C1—C2	177.25 (19)	Zn1—N5—C29—C28	−178.29 (13)
N1—C1—C2—C3	1.1 (4)	C25—N5—C29—C33	−179.36 (16)
C1—C2—C3—C4	1.3 (4)	Zn1—N5—C29—C33	0.13 (19)
C2—C3—C4—C5	−2.5 (4)	C27—C28—C29—N5	0.3 (3)
C2—C3—C4—C6	175.1 (2)	C30—C28—C29—N5	−179.94 (17)
C1—N1—C5—C4	0.9 (3)	C27—C28—C29—C33	−178.05 (17)
Bi1—N1—C5—C4	−178.59 (15)	C30—C28—C29—C33	1.7 (3)
C1—N1—C5—C9	−177.2 (2)	C29—C28—C30—C31	−2.0 (3)
Bi1—N1—C5—C9	3.2 (2)	C27—C28—C30—C31	177.8 (2)
C3—C4—C5—N1	1.4 (3)	C28—C30—C31—C32	1.3 (3)
C6—C4—C5—N1	−176.3 (2)	C30—C31—C32—C34	−177.5 (2)
C3—C4—C5—C9	179.5 (2)	C30—C31—C32—C33	−0.4 (3)
C6—C4—C5—C9	1.9 (3)	C36—N6—C33—C32	2.1 (3)
C5—C4—C6—C7	0.1 (4)	Zn1—N6—C33—C32	179.28 (13)
C3—C4—C6—C7	−177.3 (2)	C36—N6—C33—C29	−176.84 (16)
C4—C6—C7—C8	−1.9 (4)	Zn1—N6—C33—C29	0.38 (19)
C6—C7—C8—C10	−178.7 (2)	C34—C32—C33—N6	−1.4 (3)
C6—C7—C8—C9	1.6 (4)	C31—C32—C33—N6	−178.71 (17)
C12—N2—C9—C8	0.8 (3)	C34—C32—C33—C29	177.48 (17)
Bi1—N2—C9—C8	−178.14 (15)	C31—C32—C33—C29	0.2 (3)
C12—N2—C9—C5	179.3 (2)	N5—C29—C33—N6	−0.4 (2)
Bi1—N2—C9—C5	0.4 (2)	C28—C29—C33—N6	178.11 (16)
C10—C8—C9—N2	−0.8 (3)	N5—C29—C33—C32	−179.28 (15)
C7—C8—C9—N2	178.9 (2)	C28—C29—C33—C32	−0.8 (2)
C10—C8—C9—C5	−179.3 (2)	C33—C32—C34—C35	−0.3 (3)
C7—C8—C9—C5	0.4 (3)	C31—C32—C34—C35	176.9 (2)
N1—C5—C9—N2	−2.4 (3)	C32—C34—C35—C36	1.2 (3)
C4—C5—C9—N2	179.33 (19)	C33—N6—C36—C35	−1.1 (3)
N1—C5—C9—C8	176.08 (19)	Zn1—N6—C36—C35	−177.77 (15)
C4—C5—C9—C8	−2.1 (3)	C34—C35—C36—N6	−0.5 (3)
C9—C8—C10—C11	0.3 (4)		

### Hydrogen-bond geometry (Å, °)

**Table:**

*D*—H···*A*	*D*—H	H···*A*	*D*···*A*	*D*—H···*A*
C2—H2···Cl3^i^	0.93	2.82	3.588 (4)	141.
C6—H6···Cl4^ii^	0.93	2.82	3.637 (4)	147.
C10—H10···Cl5^iii^	0.93	2.80	3.707 (4)	164.
C15—H15···Cl1^iv^	0.93	2.69	3.579 (4)	160.
C25—H25···Cl2^v^	0.93	2.80	3.506 (4)	134.

Symmetry codes: (i) −*x*+1, −*y*+1, −*z*; (ii) −*x*, −*y*+1, −*z*; (iii) *x*−1, *y*, *z*; (iv) −*x*+2, −*y*+1, −*z*; (v) −*x*+1, −*y*+1, −*z*+1.
